# *In Vitro,* *in Situ *and *in Vivo* Studies on the Anticandidal Activity of *Cassia fistula *Seed Extract

**DOI:** 10.3390/molecules17066997

**Published:** 2012-06-07

**Authors:** Subramanion L. Jothy, Zuraini Zakariah, Yeng Chen, Sreenivasan Sasidharan

**Affiliations:** 1 Biological Program, School of Distance Education, Universiti Sains Malaysia, Minden 11800, Penang, Malaysia; 2 Institute for Research in Molecular Medicine (INFORMM), Universiti Sains Malaysia, Minden 11800, Pulau Pinang, Malaysia; 3 Dental Research and Training Unit, and Oral Cancer Research and Coordinating Centre (OCRCC), Faculty of Dentistry, University of Malaya, Kuala Lumpur 50603, Malaysia

**Keywords:** *Candida albicans*, *Cassia fistula* seed, anticandidal activity, scanning electron microscope, transmission electron microscope

## Abstract

*Cassia fistula* seeds have many therapeutic uses in traditional medicine practice. The present investigation was undertaken to demonstrate the anticandidal activity of the *C. fistula *seed extract at ultra-structural level through transmission electron microscope (TEM) and scanning electron microscope (SEM) observations. The effect of seed extract on the growth profile of the *Candida albicans* was examined via time-kill assays and *in vivo* efficacy of the extract was tested in an animal model. In addition, the anticandidal effect of seed extract was further evaluated by microscopic observations using SEM and TEM to determine any major alterations in the ultrastructure of *C. albicans*. The complete inhibition of *C. albicans* growth was shown by *C. fistula* seed extract at 6.25 mg/mL concentration. The time-kill assay suggested that *C. fistula* seed extract had completely inhibited the growth of *C. albicans* and also exhibited prolonged anti-yeast activity. The SEM and TEM observations carried out to distinguish the metamorphosis in the morphology of control and *C. fistula* seed extract-treated *C. albicans *cells revealed the notable effect on the outer cell wall and cytoplasmic content of the *C. albicans *and complete collapse of yeast cell exposed to seed extract at concentration 6.25 mg/mL at 36 h. The i*n vitro* time-kill study performed using the leaf extract at 1/2, 1 or 2 times of the MIC significantly inhibited the yeast growth with a noticeable drop in optical density (OD) of yeast culture, thus confirming the fungicidal effect of the extract on *C. albicans*. In addition, *in vivo* antifungal activity studies on candidiasis in mice showed a 6-fold decrease in *C. albicans *in kidneys and blood samples in the groups of animals treated with the extract (2.5 g/kg body weight). The results suggested that the *C. fistula *seed extract possessed good anticandidal activity and is a potential candidate for the development of anticandidal agents*.*

## 1. Introduction

*Cassia fistula *Linn. (Leguminosae) is cultivated widely throughout Malaysia as an ornamental and deciduous plant. It is also being cultivated in the tropics, including in the West Indies, Ceylon, China, Egypt and many other countries. As a medicinal plant it is used mainly in the West Indies, namely Dominica and Martinique [1]. “*Purging cassia*” was known in Europe in the thirteenth century and was used by the School of Medicine at Salerno [2]. In Ayurvedic medicine, this plant is used for a treatment of hematemesis, pruritus, leucoderma and diabetes. The juice of the leaf is given for erysipelas and skin diseases [3]. In Sri Lanka, this plant is used for skeletal fractures [4]. Moreover, different parts of this plant have been demonstrated to possess several pharmacological activities such as antitumor [5], antioxidant [6], hypoglycemic [7], hepatoprotective [8], antibacterial [9], hypocholesterolaemic [10], and antidiabetic [11].

Oral candidiasis is the most common opportunistic infection associated with HIV/AIDS, with up to 90% of HIV-infected individuals [12]. *Candida albicans *is the most frequent etiological strain associated with this infection and reminds the predominant strain in most of the countries. The antifungals prescribed for treating oral candidiasis in HIV-positive patients in long-term therapy has led to the development of resistance [13,14]. In this perspective, new agents from natural resources that can inhibit the growth of *C. albicans* are greatly needed and would enhance the effectiveness of the therapy. Our previous study showed that the methanol extract of *C. fistula *seed extract possessed a good anticandidal activity against *C. albicans* with minimum inhibition concentration (MIC) value of 6.25 mg/mL [15]. The present investigation demonstrates the anticandidal activity of the *C. fistula *seed extract at ultra-structural level through TEM and SEM observations at 6.25 mg/mLconcentration with *in vitro* time killing and *in vivo* animal studies.

## 2. Results

### 2.1. Time-Kill Study

**Figure 1:**
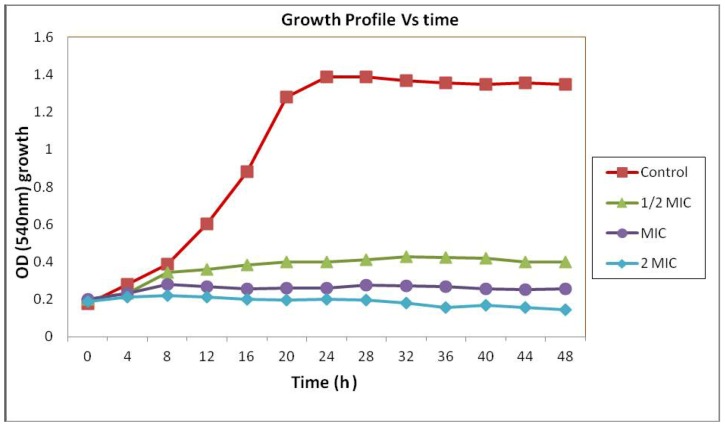
Growth profile of *Candida albicans* in Muller Hinton broth with 0 (control) and 3.13 mg/mL (1/2 MIC), 6.25 mg/mL (MIC) and 12.5 mg/mL (2 MIC) concentration of *C. fistula *seed extract.

The result obtained from the previous study indicated that *C. fistula *seed extract showed anticandidal activity. The MIC of the *C. fistula *seed extract in broth dilution method was found to be 6.25 mg/mL. Time-kill studies were performed over a period of 48 h with yeast being exposed to 1, 1/2 or 2 × MIC of the *C. fistula *seed extract. The result of the time-kill curves for *C. albicans *is shown in Figure 1. At 1/2 × MIC, *C. fistula *seedextract demonstrated a large drop in Optical Density (OD) after 16 h, which leads to the stationary phase of yeast growth compared with the control. At MIC and 2 × MIC, *C. fistula *seedextract produced absolute yeast eradication after only 4 h. The time-kill curves described above show the potency of *C. fistula *seed extract as an anticandidal agent against *C. albicans*.

### 2.2. Scanning Electron Microscopy (SEM)

**Figure 2:**
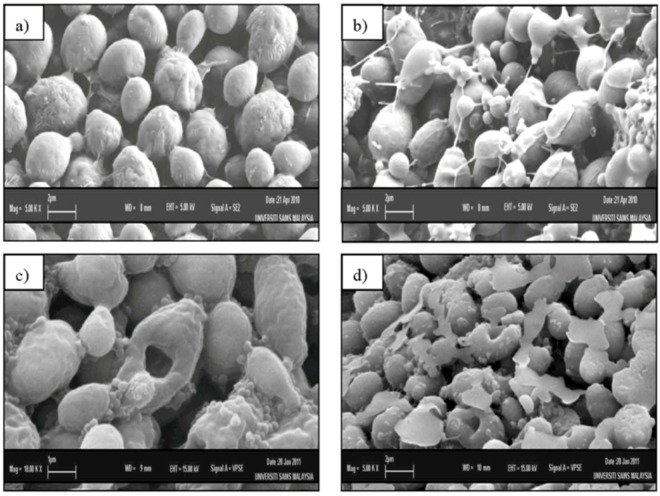
Scanning electron microscope photomicrograph of the untreated (**a**) and extract-treated (**b,c,d**) cells of *Candida albicans*.

Figure 2 shows SEM photomicrographs of the untreated and extract treated cells of *C. albicans* at various times of exposure to the crude extract of *C. fistula*. Untreated control cells (Figure 2a) showed many regular, homogeneous cells with smooth walls in appearance and some at a budding stage. After 12 h of exposure (Figure 2b), a mild effect of the extract was observed and elongated cells also appeared. The 24 h treated cells (Figure 2c) had a rough appearance with holes compared to the untreated control cells with the formation of invaginations. The surface of the *C. fistula* treated cells also appeared uniformly rough because of a well-defined wrinkling of the cell wall. Moreover, the yeast cells were present in clusters of interconnected cells. After 36 h of exposure (Figure 2d), completely collapsed or cavitated cells with rough in appearance surfaces with holes were seen. Huge amounts of vesicular material were equally found scattered in between the *C. albicans* cells. These materials were clearly seen in the 36 h treated group (Figure 2d), was most likely derived from broken cells and presented membrane-limited cytoplasmic leftovers. The remaining unbroken *C. albicans *cells showed a smooth surface as observed in untreated control cells.

### 2.3. Transmission Electron Microscope (TEM)

**Figure 3:**
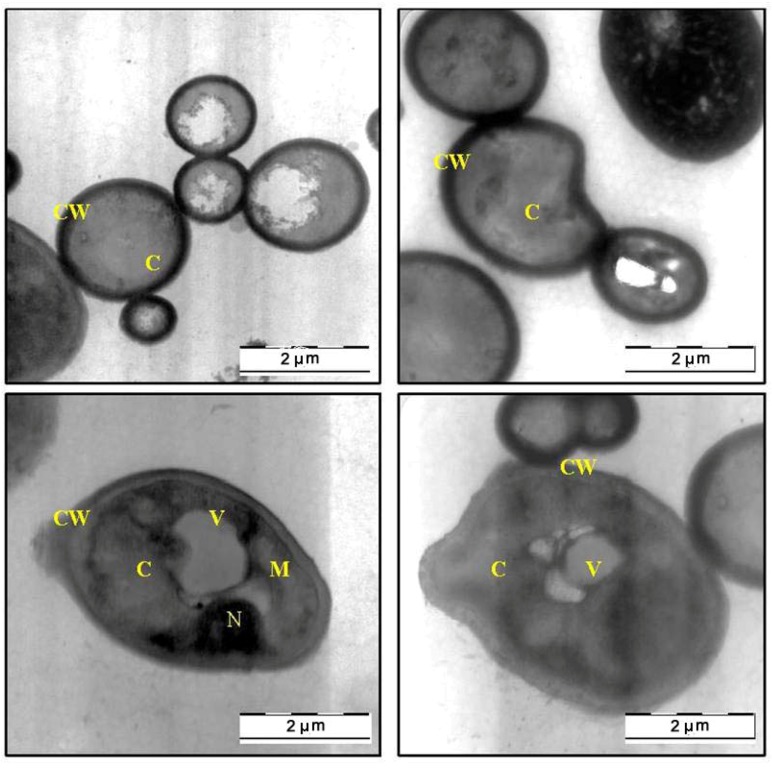
TEM micrograph of a cross-section of untreated cell of *Candida albicans *(C: Cytoplasm; CW: Cell Wall; M: Mitochondria; N: Nucleus; V: Vacuole).

**Figure 4:**
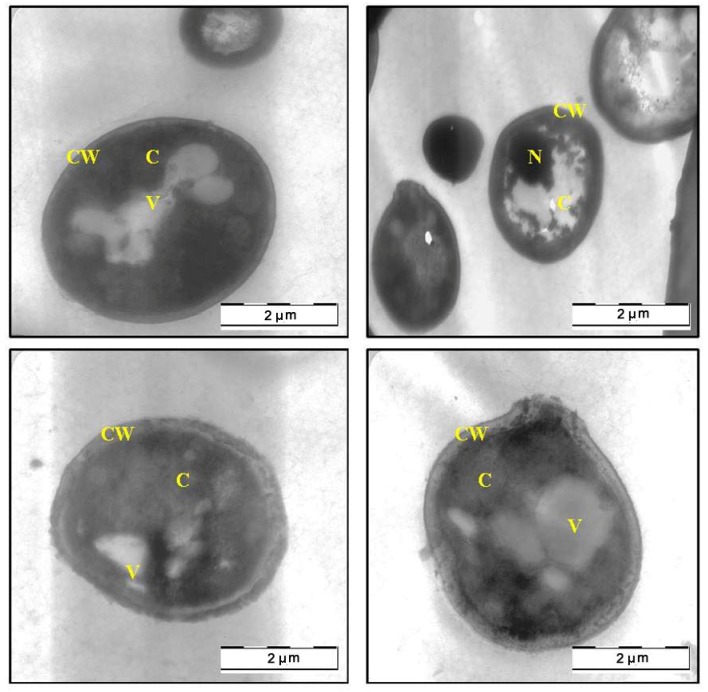
TEM micrograph of a cross-section of the extract treated cell of *Candida albicans* after 12 h (C: Cytoplasm; CW: Cell Wall; N: Nucleus; V: Vacuole).

**Figure 5:**
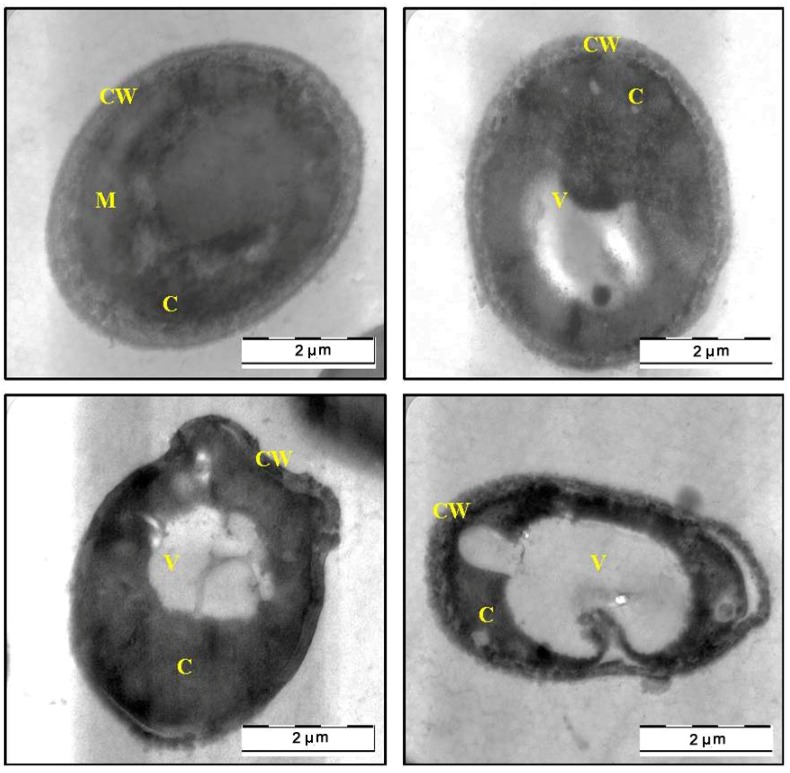
TEM micrograph of a cross-section of the extract treated cell of *Candida albicans* after 24 h (C: Cytoplasm; CW: Cell Wall; M: Mitochondria; V: Vacuole).

**Figure 6:**
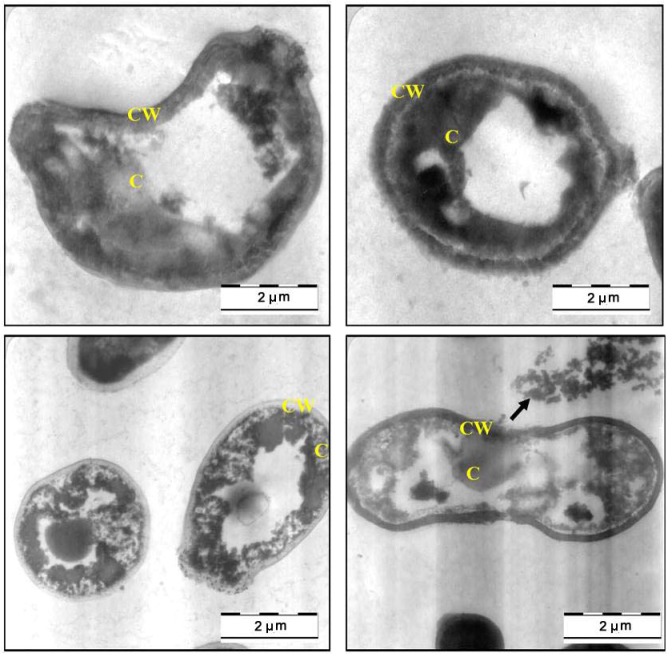
TEM micrograph of a cross-section of the extract treated cell of *Candida albicans* after 36 h (C: Cytoplasm; CW: Cell Wall).

The SEM findings, suggested that the methanol extract of *C. fistula* can caused severe alteration in the morphology of *C. albicans*. Further evidence of anticandidal activity was obtained from a TEM study. Figure 3 shows the TEM photomicrographs of the longitudinal and transverse sections of the untreated control cells of *C. albicans*. The cytoplasm of *C. albicans* cells appeared homogeneous containing a nucleus, vesicle and mitochondria, surrounded by a defined cell membrane and regular cell wall with a clear periplasm region. After 12 h of exposure to the *C. fistula* extract (Figure 4), the cell was very densed with the vesicles and membranous bodies dispositioned within the cell. After 24 h of exposure (Figure 5), the cells showed shrinkage of the protoplast, disruption of the cytoplasmatic membrane and notable alterations in the cell wall. The cytoplasmic volume decreased and the cell membrane invaginated with notable structural disorganization within the cell cytoplasm. It seems that the extract induced dysfunctions of the cell membrane. Figure 6 shows the significant effect of the extract on the yeast cells after 36 h of exposure. All the inner organelles were completely discomposed and even cell membrane and wall were deeply affected and look like undulant. Yeast cells were found collapsed which followed by an outflow of the cytoplasmic component (Figure 6).

### 2.4. *In Vivo* Antifungal Activity

Table 1 shows the mean of CFU/g organ and CFU/mL of blood from the two groups. In Group 2 animals that received a 2.5 g/kg body weight dose of the plant extract followed by inoculation of *C. albicans*, a significant reduction (*p* < 0.05) in CFU was observed in kidney and blood samples studied. A 6-fold difference was present in the kidney and blood samples of the treated group compared with those of the control group. 

**Table 1:** Effect of methanolic extract of *Cassia fistula* seed on *Candida albicans *recovered from kidney and blood of mice.

Group	Kidney (CFU/g)	Blood (CFU/mL of blood)
**Group 1 (control) i.v. Candida + i.p. PBS **	2.19 × 10^5^ ± 14,322	2.76 × 10^5^ ± 11,725
**Group 2 (curative) i.v. Candida + i.p. Extract**	3.87 × 10^4^ ± 73 ^a^	4.68 × 10^4^ ± 47 ^a^

All values are colony-forming units (CFU/g organ or CFU/mL of blood) expressed as mean ± standard error of mean of 10 determinations. ^a^*p *< 0.05 compared with control (Student *t*-test).

## 3. Discussion

*C. fistula* seed extract exhibited a favorable anti-yeast activity against *C. albicans *with a MIC value of 6.25 mg/mL [15]. The MIC value was defined as the lowest extract concentration that completely inhibited visible growth of *C. albicans*. Effectiveness of anti-yeast activity inversely correlated with their MIC values. Kuete *et al. *[16] reported that the extract having activities where the MIC values are below 8 mg/mL are considered to possess some antimicrobial activity. Time-kill assay was utilized in this study to verify MIC findings and to evaluate the ability of *C. fistula *seedextract to eliminate *C. albicans* growth *in vitro*. In the case of one- and two-times MIC concentrations, the extract inhibited the yeast growth within 4 h and subsequent regrowth was not seen. 

The time-kill assay suggested that *C. fistula *extract completely inhibited the growth of *C. albicans* and it also exhibited prolonged anti-yeast activity against the *C. albicans* as determined by time-kill curves. These results hypothesised that the phytochemicalsof *C. fistula *seed extract attacked the *C. albicans *cells and caused higher cell damage. To verify this hypothesis, *C. albicans* cells (untreated and *C. fistula *seed extract treated) were observed through different microscopic techniques. In this study, the methanolic extract of *C. fistula *seed was shown to have significant anticandidal activity in an animal model when given by intraperitoneal injection followed by inoculation of *C. albicans*, indicating a strong antifungal activity.

In this communication, we were especially interested in the mophogenesis of the *C. albicans *cells challenged by *C. fistula* seed extract. *In vitro* observation of anticandidal activity of *C. fistula* seed extract in this study was further evaluated by *in situ* microscopic method to postulate the possible mechanisms of action in *C. albicans*. The morphological observation of *C. albicans* cells treated with plant extract by using electron microscope is considering a gold standard technique to study the *in situ* anticandidal activity. 

In this study, SEM and TEM observations were utilized to study the anticandidal activity of *C. fistula* seed extract. The SEM and TEM methods are advantageous over several other microscopic methods as they are 3-dimentional and almost the whole cell of the specimen is sharply focused [17]. The microscopic examination of *C. albicans *using SEM showed that the cells treated with *C. fistula *seed extract decreased in size, appeared irregular in shape with cell wall modifications and clear depressions on the cell surface with holes. Also, TEM observation showed irregular cell walls, ruptured cell membranes, unclear periplasm and dense cytoplasm without differentiated features. Such modifications may be related to the interference of the seed extract components with enzymatic reactions of wall synthesis which affects yeast morphogenesis and growth. The cytoplasmic membranes of yeast provide a barrier to the passage of small ions such as H^+^, K^+^, Na^+^ and Ca^2+^ and allow cells and organelles to control the entry and exit of different compounds. This permeability barrier role of cell membranes is integral to many cellular functions, including the maintenance of the energy status of the cell, other membrane-coupled energy-transducing process, solute transport, regulation of metabolism and control of turgor pressure [18,19,20]. When cells of *C. albicans *were incubated with *C. fistula *seed extract, the leakage of ions from both treated and untreated cells were nearly the same within 12 h. This implied that the extract had a little effect on the cells or their membranes during the first 12 h. But within 12 to 36 h, significant increase in K^+^, Ca^2+^ and Mg^2+^ leakage from treated cells might have occurred. For these ions (K^+^, Ca^2+^ and Mg^2+^) to be released from the cells, some changes in the cell membrane may have occurred after treated with the extract. The exposure to the extract increased the permeability of plasma membrane by disrupting the permeability barrier of cell membrane structures accompanied by the loss of chemiosmotic control [21]. Hence, the effect of seed extract, on the *C. albicans* may attribute to the disruption of fungal membrane which was observed by SEM as damaged membrane accompanied by an important surface alteration. 

The results from this study also agree with the suggestion by Prashar *et al.* [22] that the release of ions is not only based on their size and/or due to formation of holes or lesions of lipid bilayer of the plasma membrane of *C. albicans* but with various steps. Actually, the anticandidal action of *C. fistula* seed extract may happen in two steps. The first step involves the passive entry of the seed extract into the plasma membrane in order to initiate membrane disruption. The second stage is the accumulation of *C. fistula* seed extract in the plasma membrane resulting in the inhibition of cell growth [22]. Hence, *C. fistula* has the potential compounds with the above mentioned possible mechanisms which deserved further detailed studies.

## 4. Experimental

### 4.1. Plant Collection

Fresh pods of *Cassia fistula* were collected from various areas in Universiti Sains Malaysia, Penang in November, 2010 and authenticated by the botanist of the School of Biological Sciences at Universiti Sains Malaysia where the herbarium sample was deposited. The sun-dried pods are cut open and the seeds were removed from the pods. The seeds were then washed thoroughly and rinsed with tap water and dried in oven at 60 °C for three to four days. Then the dried seeds were homogenized to a fine powder and stored in airtight bottles. 

### 4.2. Preparation of the Crude Extracts

The seed powder was sequentially extracted with methanol by adding approximately 100 g of the dried sample (in fine powder form) to 400 mL methanol. The extraction was carried out at room temperature by soaking for 7 days with intermittent stirring during the first day. The extracts were filtered through clean muslin cloth and the process of extraction was repeated again for a second time by adding another 400 mL of methanol to the sample residue. The filtrate from each extraction was combined and concentrated under vacuum by rotary evaporator (Büchi, Switzerland) at 40 °C to 50 °C in order to evaporate the excess methanol solvent and until a dark green methanol extract was produced. Then concentrated extract was poured in glass Petri plates and brought to dryness at 60 °C in the oven until a paste-like mass was obtained. Then paste form extract was sealed in Petri plates and stored at room temperature (RT). The crude extract was prepared by diluting the paste in methanol and stored in air-tight bottles at 4 °C for further studies.

### 4.3. Microorganism

*Candida albicans *strain (local clinical isolate) was used as the test organism and was obtained from a laboratory stock culture. The yeast was cultured on Sabouraud dextrose agar at 30 °C for 24 h. The stock cultures were maintained on Sabouraud dextrose agar slants at 4 °C.

### 4.4. Growth Profile of *C. albicans* in the Presence of *C. fistula* Seed Extract

The Minimum Inhibitory Concentration (MIC) of extract against *C. albicans* was determined by using broth dilution method. In order to assess the anticandidal activity with MIC, 1/2 MIC, and 2 MIC concentration over time, growth profile curves were plotted [23]. A 16 h culture was harvested by centrifugation, washed twice with phosphate saline, and resuspended in phosphate saline. The suspension was adjusted using the McFarland standard and was then further diluted in phosphate saline to achieve approximately 10^7^ CFU mL^−1^. *C. fistula *seed extract was added to aliquots of 25 mL Mueller-Hinton broth (MHB) in a 50-mL Erlenmeyer flask (37 °C) to achieve a concentration of 0 (control), 6.25 mg/mL (MIC), 3.13 mg/mL (1/2 MIC), and 12.5 mg/mL (2 MIC) after addition of the inocula. One ml of inocula was added to all Erlenmeyer flasks. Finally, 1 mL portion was removed and the growth of *C. albicans *was monitored using this portion by measuring optical density at 540 nm using UV-Visible spectrophotometer (UV-9100; Ruili Co., Beijing, China). The growth of *C. albicans *was measured every 4 h for 48 h continuously by the above method.

### 4.5. Scanning Electron Microscopy (SEM) Observation

Scanning electron microscope observations were carried out on *C. albicans* cells. One milliliter of *C. albicans* cell suspension at concentration 1 × 10^6^ cells per milliliter was inoculated on Sabouraud Dextrose Agar plate and then incubated at 30 °C for 6 h. One milliliter of 6.25 mg/mL, *C. fistula* seed extract was dropped onto inoculated agar and further incubated for 36 h at the same temperature. The cells which were not treated with extract were used as a control. A small block of yeast containing agar was withdrawn from plate at 0, 12, 24 and 36 h placed on planchette and fixed for scanning (LEO SUPRA 50 VP Field Emission SEM, Carl Zeiss, Oberkochen, Germany) [24]. The SEM observation was done under the following analytical condition: L = SE1, EHT = 5.00 kV and WD = 8 mm to study the effect of extract on *C. albicans* cells. 

### 4.6. Transmission Electron Microscope (TEM) Observation

Transmission Electron Microscope (TEM) observations were also carried out on *C. albicans* cells. The preparation procedure of *C. albicans* cells on plates was similar as described in SEM section. The TEM analyses were performed [24] on sample which were harvested at same hours (0, 12, 24 and 36 h) and fixed in McDowell-Trump fixative prepared in 0.1 M phosphate solution (pH 7.2), rinsed in buffer for three times, postfixed in 1% of osmium tetroxide in buffer for 2 h at 4 °C then the sample was serially dehydrated with 50%, 75%, 95% and 100% ethanol respectively and embedded in Epon- Araldite resin for making the blocks of the cells. The resin with the embedded *C. albicans* cells were cut into ultra thin sections in the ultramicrotomy process. Finally, the ultra-thin sections of the cells were stained with 2% uranyl acetate and lead citrate and observed under a Transmission Electron Microscope (TEM) (LIBRA 120-ZEISS, Oberkochen*, *Germany).

### 4.7. *In Vivo* Antifungal Activity

#### 4.7.1. Laboratory Animals

Swiss albino mice (male) weighing between 25 and 35 g were used. The cages with the mice were placed in a room (temperature 26 ± 2 °C) with controlled cycles of 12 h of light and 12 h of darkness; light went on at 7 am and relative humidity was 45–55%. Water and food were provided to animals *ad libitum*. The experimental protocols were approved by the Institutional Animal Ethics Committee (IAEC) of the School of Pharmaceutical Sciences, Universiti Sains Malaysia. Experiments were conducted in accordance with the internationally accepted principles for laboratory animal use and care (USM/ Animal Ethics Approval/ 2010/ (59)(262)).

#### 4.7.2. Antifungal Assay

The standard intravenous (i.v.) inoculation of *C. albicans *used in this study was 1 × 10^7^ viable cells/mL PBS, of which 0.1 mL was injected into the lateral tail vein of mice [25]. Animals were divided into two groups of 10 mice each and received treatment as described in Table 2. All mice were killed by cervical dislocation on day 5 after i.v. *C. albicans *inoculation.

**Table 2:** Details of experimental groups.

Group	Treatment
**Group 1 (control)**	i.v. *C. albicans*: 24 h gap, followed by treatment with PBS (i.p. once daily for 3 days)
**Group 2 (curative)**	i.v. *C. albicans*: 24 h gap, followed by treatment with *Cassia fistula *seed extract, 2.5 g/kg body weight (i.p. once daily for 3 days)

i.v., intravenous; i.p. intraperitoneal; PBS Phosphate buffer solution.

The kidneys of each animal were removed aseptically, and 0.1 mL of blood was withdrawn from the renal artery and 0.1 mL of heparin (25 U/mL), as an anticoagulant was added into the blood sample. The kidneys were then, placed in sterile centrifuge tubes and homogenized in 5 mL of sterile PBS. Aliquots from each homogenate and blood samples were serially diluted, plated on Sabouraud dextrose agar plates, and incubated at 37 °C for 24 h. All cultures were done in triplicate. The colonies were then enumerated and the colony forming units (CFU) were calculated per gram of organ and per mL of blood sample, respectively. The numbers of colonies from the control and the test group were compared using the *t*-test run on the software SPSS for Windows.

## 5. Conclusions

To our knowledge, this is the first detailed *in vitro *and *in vivo *study on the anticandidal effects of *C. fistula *seed extract against *C. albicans* and it revelas that it may be a promising remedy for the development of anticandidal agents in the future. Planned further studies on the activity-directed fractionation for the isolation of respective pure compounds may result in interesting outcomes.
